# Comparison of risk factors for Methicillin Resistant Staphylococcus aureus (MRSA) colonization in healthy newborns, born to mothers with and without MRSA colonization

**DOI:** 10.12669/pjms.40.1.7703

**Published:** 2024

**Authors:** Sara Malik, Muhammad Faheem Afzal, Muhammad Haroon Hamid

**Affiliations:** 1 Sara Malik, MD Senior Registrar, University of Child Health and Sciences, Children Hospital, Lahore, Pakistan; 2 Muhammad Faheem Afzal, FCPS, FCPS (Pediatric Infectious Disease), MHPE Professor Pediatric Medicine, Ameer ud Din Medical College PGMI / Lahore General Hospital, Lahore, Pakistan; 3 Muhammad Haroon Hamid, FCPS (Pediatrics) MRCP, FRCP Chairman/Professor Pediatric Medicine, Chief executive Officer, King Edward Medical University, Mayo Hospital, Lahore, Pakistan

**Keywords:** Risk factors, Newborn, Maternal vaginal and nasal Methicillin Resistant Staphylococcus aureus colonization

## Abstract

**Objective:**

To compare the risk factors for MRSA colonization in healthy newborns, born of mothers with and without MRSA colonization.

**Methods:**

This case control study was conducted in post-natal unit of Lady Willingdon Hospital, affiliated with King Edward Medical University/Mayo Hospital, Lahore from January to June 2017. The vaginal and anterior nares swabs for MRSA culture were collected from mothers within six hours before planned delivery and the neonatal anterior nares swabs for MRSA culture were taken within one hour of birth. All the samples were cultured in Paediatric Microbiology laboratory in Mayo Hospital. Data were analyzed through SPSS 20.0 and logistic regression was applied for risk factors analysis.

**Results:**

Out of total 80 mothers and their newborns, 15 (18.75%) mothers and 16 neonates (20%) were MRSA colonized. The frequency of MRSA colonization in mothers’ anterior nares and vaginal swab was 17.5% and 1.25% respectively. The significant risk factors were prolonged rupture of membranes for >18 hours (p-value 0.02, odds ratio 11.85, 95% CI 1.41-99.3), birth weight <2500 grams (p-value 0.01, odds ratio 5.39, 95% CI 1.35-21.4), history of presence of meconium (p-value 0.006, odds ratio 7.30, 95% CI 1.78-29.8). The non-significant factors were age of mother (p-value 0.682, odds ratio 0.765, 95% CI 1.0.212-2.76), parity (p-value 0.185, odds ratio 3.82, 95% CI 0.46-31.66) , gestation (p-value 0.615, odds ratio 0.797, 95% CI 0.714-0.89) , mode of delivery (p-value 0.576, odds ratio 0.543, 95% CI 0.062-4.76), sex of baby (p-value 0.546, odds ratio 0.683, 95% CI 0.196-2.37) and presentation of baby at birth (p-value 0.47, odds ratio 0.795, 95% CI 0.71-0.89)

**Conclusion:**

The presence of meconium, prolonged rupture of membranes and low birth weight were the significant risk factors for MRSA colonization in healthy new-borns, born to mothers with and without MRSA colonization.

## INTRODUCTION

Neonatal infections can lead into wide spectrum of illness in neonatal intensive care units (NICUs). Staphylococcus aureus is a commensal and an opportunistic pathogen which often exists as part of the normal flora on human skin and mucosal surfaces.^1^,^2^ It is the second most common cause of sepsis in very-low birth weight neonates admitted to neonatal intensive care units (NICUs).^3^ Preterm infants are also at high risk for Staphylococcus aureus colonization, a potential risk factor for subsequent infection.^4^ Methicillin-resistant Staphylococcus aureus (MRSA) is a group of gram-positive cocci that is resistant to methicillin. MRSA carriers have a higher risk for developing clinical infections.^5^,^6^

The frequency of MRSA colonization in NICU setting has been reported to be 5.2%.^6^ The prevalence of MRSA carriage in vaginal sites of pregnant women is 1.7% and 5.7% in anterior nares showed the neonatal incidence of MRSA carriage is 0.8%.^7^,^8^ There are many risk factors for MRSA colonization in neonates including low birth weight, prematurity and multiple gestation.^6^ Advanced maternal age and lack of access to proper healthcare facilities during pregnancy are the leading risk factors for maternal colonization. Since MRSA colonization from healthcare workers or household contacts is possible, there should be identification of all environmentally and maternal derived risk factors.^8^

This study has been designed to determine the risk factors for MRSA colonization in healthy neonates, born to mothers with and without MRSA colonization. According to our knowledge, no data is available in Pakistan on risk factors for MRSA colonization in healthy newborns. The objective of this study was to compare the risk factors for the MRSA colonization in healthy newborn, born to mothers with and without MRSA colonization.

## METHODS

This case control study was conducted in the post-natal unit of Lady Willingdon Hospital, Lahore, affiliated with Department of Pediatrics, King Edward Medical University, Lahore from January-June, 2017. This hospital has an obstetrics unit; therefore, neonates (~500–550 annually) are admitted to this post-natal unit having level III care.Total 80 paired samples were enrolled by non-probability convenient sampling (estimated by using 10% level of significance, 90% power of test with expected percentage with MRSA as 26%^9^ and MRSA colonization as 8.4%).^7^ Isolation of MRSA from anterior nares (for newborn) and from anterior nares and/ or vagina (for mothers) without clinical evidence of infection was considered as MRSA colonization in healthy newborn.

### Ethical Approval

The study was approved by institutional review board (ERC approval# 7699-7703/REG/KEMU/2016 and 05/08/2016). Informed and written consent was taken from father or mother.

### Inclusion criteria

Newborns of either sex, delivered by either route and needing no admission and resuscitation within one hour of birth, whereas mother within six hours prior to planned delivery were included in the study.

### Exclusion criteria

Any mother on antibiotic for presumed infection irrespective of MRSA within 48 hours prior to delivery and preterm newborns (<37 weeks of gestation) were excluded.

The maternal risk factors for MRSA (maternal age, multiple gestation (≥2 embryos in uterus), parity, prolonged rupture of membrane and mode of delivery) and neonatal risk factors for MRSA (birth weight, baby gender, mode of delivery, presentation of baby (vertex/breech) at delivery, presence of meconium) were recorded. The vaginal and anterior nares swabs for MRSA culture were collected from mothers within six hours before planned delivery and the neonatal anterior nares swabs for MRSA culture were taken within one hour of birth. In case of multiple gestations, anterior nares swab was taken from first baby. Swab was removed carefully and put into the transport media Cary Blair Media immediately and sent to the Pediatric Microbiology laboratory. Swabs were streaked onto a differential media. Each specimen was cultured on blood agar and MacConkey agar which is a selective medium with 7-9% NaCl that allows *S*. aureus to grow, producing yellow-colored colonies as a result of mannitol fermentation and subsequent drop in the medium’s pH at 35°-37°C for 18-24 hours. All plates were examined for yellow-colored colonies consistent with MRSA. If negative, the plates were re incubated for another 24 hours. The antibiotic susceptibility testing was done on Baird-Parker agar containing methicillin. The plates were incubated aerobically at 37°C for 24 hours, after which the zones of inhibition were measured. Resistance to methicillin was considered as methicillin resistance. Mupirocin was given to MRSA positive newborn as topical nasal ointment, that was applied to inner parts of nose, twice a day for five days.

Data was analyzed through SPSS 20.0. Quantitative variables (maternal age, birth weight, parity) were presented as mean±S.D. Qualitative variables (baby gender, multiple gestation, prolonged rupture of membrane, presentation of baby at delivery, presence of meconium and mode of delivery) were presented as frequency and percentages. Logistic Regression was applied for analysis of risk factors in both the groups. (Positive for MRSA and negative for MRSA). P-value <0.05 was taken as significant.

## RESULTS

There were 63 (78.8%) mothers of age less than 30 years. Out of 80 mothers, 75 mothers (93.8%) had no history of prolong rupture of membranes >18 hours. There were 66 multiparous mothers (82.5%). There were 25 male newborns (31.2%) and 55(68.8%) females. Seventy- two (90%) mothers had vaginal delivery. There were 61 babies (76.2%) of birth weight <2500 grams. Seventy eight (97.5%) neonates had cephalic presentation while two babies had breech presentation (2.5%). There were 16 babies (20%) having history of presence of meconium. The frequency of MRSA colonization in mothers’ anterior nares and vagina was 17.5% and 1.25% respectively. The frequency of anterior nares MRSA colonization in neonates was 20%.

On logistic regression, history of prolong rupture of membranes >18 hours (p-value 0.021, odds ratio 7.15, 95% CI 1.08-47.18), birth weight <2500 grams (p-value 0.036, odds ratio 3.37, 95% CI 1.04 -10.86) and history of presence of meconium (p-value 0.006, odds ratio 7.30, 95%CI 1.41-15.99) were found to be the risk factors for MRSA colonization in babies, (Table-I & II).

**Table-I:** Comparison of neonatal MRSA colonization with maternal vaginal MRSA colonization (n=80)

Mother’s MRSA status		Baby’s MRSA status		Total	p-value	Odds ratio	95% Confidence interval

		Positive	Negative				
Vaginal swab	Positive	0 (0%)	1(100%)	1(100%)	0.615	1.25	1.122-1
	Negative	16(20%)	63(80%)	79(100%)			
Nasal swab	Positive	6(42%)	8(58%)	14(100%)	0.019	4.2	1.20-14.72
	Negative	10(15%)	56(85%)	66(100%)			
Age groups (years)	< 30	12(19%)	51(81%)	63(100%)	0.682	0.765	0.212-2.76
	≥30	4(23%)	13(77%)	17(100%)			
PROM	Yes	3(60%)	2(40%)	5(100%)	0.021	7.15	1.08-47.18
	No	13(17%)	62(83%)	75(100%)			
Parity	>1	15(22%)	51(78%)	66(100%)	0.185	3.82	0.46-31.66
	1	1(7%)	13(93%)	14(100%)			
Gestation	Single	16(20%)	63(80%)	79(100%)	0.615	0.797	0.714-0.891
	Multiple	0 (0%)	1(100%)	1(100%)			
Mod of delivery	C-section	1 (12%)	7(88%)	8(100%)	0.576	0.543	0.062-4.76
	SVD	15(21%)	57(79%)	72(10%)			
Baby’s gender	Male	4(16%)	21(84%)	25(100%)	0.546	0.683	0.196-2.37
	Female	12(22%)	43(78%)	55(100%)			
Birth weight(G)	≥2500	7(36%)	12(64%)	19(100%)	0.036	3.37	1.05-10.86
	<2500	9(15%)	52(85%)	61(100%)			
Presentation of baby	Cephalic	16(21%)	62(78%)	78(100%)	0.47	0.795	0.71-0.89
	Breech	0(0%)	2(100%)	2(100%)			
Presence of meconium	Yes	7(44%)	9(56%)	16(100%)	0.008	4.75	1.41-15.99
	No	9(14%)	55(86%)	64(100%)			

**Table-II:** Multivariate Analysis.

	Β	Df	p-value	Odds ratio	95.0% C.I for Odds ratio	

					Lower	Upper
Prolong rupture of membrane >18 hours	2.473	1	.023	11.854	1.414	99.386
Birth weight < 2500 g	1.686	1	.017	5.397	1.356	21.478
Presence of meconium	1.989	1	.006	7.306	1.786	29.879
Constant	-4.381	1	.000	0.013		

On comparison of risk factors, the presence of meconium was found in five MRSA colonized babies, born to MRSA colonized mothers and in two MRSA colonized neonates, born to mothers without MRSA colonization (p-value <0.001, odds ratio 5.61, 95% CI 2.34-13.44). Similarly, history of prolonged rupture of membranes >18 hours was found in two MRSA colonized babies, born to MRSA colonized mothers and in one MRSA colonized neonate, born to mother without MRSA colonization (p -value 0.043, odds ratio 2.95, 95% CI 1.24- 7.01). Birth weight < 2500 grams was found in six MRSA colonized babies, born to MRSA colonized mothers and in three MRSA colonized neonates, born to mothers without MRSA colonization (p -value 0.004, odds ratio 5.44, 95% CI 1.49- 7.57), (Table-III).

**Table-III:** Comparison of risk factors in new-borns, born of mothers with and without MRSA colonization.

	MRSA status of Mothers						

	Positive (N=15)		Negative (N=65)		p-value	Odd ratio	95% CI

	MRSA status of Babies		MRSA status of Babies				

	Positive (n=6)	Negative (n=9)	Positive (n=10)	Negative (n=56)			
PROM	2	1	1	1	0.043	2.95	1.24-7.01
BW<2500gm	6	4	3	48	0.004	5.44	1.49-7.57
Meconium positive	5	4	2	5	<0.001	5.61	2.34-13.44

PROM (prolong rupture of membranes).

## DISCUSSION

Among 80 mothers in this study, 15 (18%) were colonized with MRSA according to nasal or vaginal swabs. Vaginal MRSA was detected in one case (1.25%). In the current study, these 15 MRSA-positive pregnant women are categorized as the MRSA positive group, and the remaining 65 pregnant women as the MRSA negative group. The frequency of vaginal MRSA colonization (1.25%) in mothers in the current study is similar to Ogura J, et al.^10^ that showed 55 pregnant women (6.1%) were colonized with MRSA according to nasal or vaginal swabs. Vaginal MRSA was detected in nine cases (1.0%). Lin et al^7^ results showed 1.7% and Elliyas et al.^11^ study had 2.9% vaginal MRSA colonization in pregnant women. In the current study, the reasons for lower vaginal MRSA colonization in mothers might be due to geographical differences, maintenance of vaginal hygiene by mothers, maintenance of hand hygiene of health care workers and exclusion of mothers taking any treatment for documented evidence of MRSA infection within 48 hours prior to delivery.

Our study, Lin et al.^7^ ,Ogura et al.^10^, and Elliyas et al.^11^ showed that maternal age is not significantly associated with MRSA colonization but explained that women aged 30 or more years were more likely to be colonized by MRSA colonization than 20–24 years old women.^12^,^13^ The mode of delivery did not affect neonatal MRSA colonization in our study, comparable with Lin et al^7^ and Elliyas et al.^11^ Our study observed that multiple gestation status is not related with MRSA colonization in neonates but Ogura et al^10^ explained that multi parity was a risk factor for maternal MRSA colonization (OR 2.35, 95% CI 1.25–4.42). Elliyas et al.^11^ explained that there was a statistically significant relationship between parity and vaginal colonization (p=0.01). The odds predicted that multigravida is 1.4 times more colonized than primigravida with OR 1.399 (95% CI 1.064-1.84).

Birth weigh < 2500 grams is significantly associated with MRSA colonization. The current study’s result is well supported by Dong Y et al.^14^ Similarly Schuetz et al.^15^ showed that neonatal MRSA colonization rate was increased with median birth weight 1320 g (IQR 925 g–1913 g, p = 0.004).

Meconium is sterile at birth and then is colonized post natally. This correlation is weak however, so there are likely other factors that also impact the bacterial load, such as prolonged rupture of membranes, mode of birth, and environmental exposure.^16^ The history of presence of meconium is another risk factor from our study (p-value 0.006, odds ratio 7.30, 95% CI 1.78-29.8) that is comparable to Gabrriel et al.^17^ If there is prolonged / obstructed labour or prolong rupture of membranes, pathogenic bacteria colonizes the birth canal.^18^ MRSA colonization in neonates was associated with maternal history of prolonged rupture of membranes > 18 hours as explained by Lin et al. study.^7^

### Limitations

The study was conducted in a single center with limited sample size, un-documentation of MRSA status of health care workers, and limited duration of sample collection. Based on mentioned limitations, results of this study cannot be generalized. Large multicenter studies with larger sample size, and screening of health care workers for MRSA may be conducted.

## CONCLUSION

The presence of meconium, prolonged rupture of membranes for more than 18 hours and birth weight < 2500 g were the significant risk factors in healthy new-borns, born to mothers with and without MRSA colonization. The preventive strategies should be implemented for MRSA colonization reduction, subsequently leading to prevention of MRSA infection.

### Authors contribution

**SM, MFA:** Conceived, designed and editing of manuscript. **SM, MFA:** Data collection and manuscript writing. **SM:** Statistical analysis and responsible for the accuracy of the work. **SM, MFA** and **MHH:** Review and final approval of manuscript.
